# Proteomic analysis of total cellular proteins of human neutrophils

**DOI:** 10.1186/1477-5956-7-32

**Published:** 2009-08-31

**Authors:** Gisele G Tomazella, Idalete da Silva, Helen J Laure, José C Rosa, Roger Chammas, Harald G Wiker, Gustavo A de Souza, Lewis J Greene

**Affiliations:** 1 Centro de Química de Proteínas, Centro Regional de Hemoterapia e Faculdade de Medicina de Ribeirão Preto, Universidade de São Paulo, 14049-900, Ribeirão Preto, Brasil; 2 Departmento de Bioquímica, Disciplina de Biologia Molecular, Universidade Federal de São Paulo, São Paulo, Brasil; 3 Laboratório de Oncologia Experimental LIM/24, Faculdade de Medicina, Universidade de São Paulo, São Paulo, Brasil; 4 Section for Microbiology and Immunology, The Gade Institute, University of Bergen, Bergen, Norway

## Abstract

**Background:**

Neutrophils are the most abundant leukocytes in peripheral blood and represent one of the most important elements of innate immunity. Recent subcellular proteomic studies have focused on the identification of human neutrophil proteins in various subcellular membrane and granular fractions. Although there are relatively few studies dealing with the analysis of the total extract of human neutrophils, many biological problems such as the role of chemokines, adhesion molecules, and other activating inputs involved in neutrophil responses and signaling can be approached on the basis of the identification of the total cellular proteins.

**Results:**

Using gel-LC-MS/MS, 251 total cellular proteins were identified from resting human neutrophils. This is more than ten times the number of proteins identified by an initial proteome analysis of human neutrophils and almost five times the number of proteins identified by the first 2-DE map of extracts of rat polymorphonuclear leukocytes. Most of the proteins identified in the present study are well-known, but some of them, such as neutrophil-secreted proteins and centaurin beta-1, a cytoplasmic protein involved in the regulation of NF-κB activity, are described here for the first-time.

**Conclusion:**

The present report provides new information about the protein content of human neutrophils. Importantly, our study resulted in the discovery of a series of proteins not previously reported to be associated with human neutrophils. These data are relevant to the investigation of comparative pathological states and models for novel classes of pharmaceutical drugs that could be useful in the treatment of inflammatory disorders in which neutrophils participate.

## Background

Neutrophils (polymorphonuclear leukocytes, PMNs) are highly specialized blood cells which play an important role in innate immunity [1]. They constitute the first line of defense of the organism and are the most abundant leukocytes in human peripheral blood [2]. Neutrophils normally circulate in the peripheral blood, where they respond quickly to danger signals such as invading pathogens. Initially in the state of alert, when they detect other activation factors [3], PMNs pass to the state of complete activation and develop a finely modulated response [4], migrating from the circulatory system through the endothelial cell layer to the sites where chemical signals have been emitted [3]. At the target site, the activated PMNs attack microorganisms and debris by phagocytosis or by releasing a combination of reactive oxygen species (ROS), enzymes and antimicrobial peptides [5].

Although PMNs are able to instantly kill pathogens in innate immunity, neutrophils have the potential to orchestrate adaptive immune responses. They release pro-inflammatory chemokines and cytokines, which also attract and stimulate T cells and dendritic cells (DCs) [6]. In the presence of inflammation, PMNs can travel from the site of infection to the nearest lymph node [7], where they undergo apoptosis and are taken up by DCs. As a consequence, DCs can present PMN-derived antigens to T cells, instructing the responses of Th1 and Th2, and acquire antigen-presenting functions themselves [8] or transfer antigens directly to DCs [9].

Recent studies of subcellular proteomes have focused on the identification of human neutrophil proteins in various subcellular fractions, such as granules [10], cytoskeleton [11], secretory vesicles and plasma membrane [12,13]. There are relatively few studies dealing with the analysis of total cellular proteins of human neutrophils. Some have reported the partial characterization of resting human PMN proteins after separation on 2-DE gels [14]. Specifically, the role of chemokines, adhesion molecules, and other activating inputs involved in neutrophil responses as well as signaling pathways are the subjects of ongoing investigations [15]. We have applied shotgun proteomics to this problem as also done by Uriarte et al [13] who determined the subcellular proteome of secretory vesicles and plasma membrane of human neutrophils.

Using a Gel-LC-MS/MS approach we identified 251 proteins, complementing and extending the report of Castro et al [14], who used 2-DE electrophoresis and MALDI-TOF mass spectrometry to characterize the initial proteome profile of resting human neutrophils. Nonetheless, our results are also supported by the previous findings of subcellular proteomic studies. Some of the proteins identified here are reported for the first time in human neutrophils, such as apoptosis-associated speck-like protein containing a caspase activation and recruitment domain (CARD) and centaurin beta-1.

We believe that the precise understanding of the protein composition of human neutrophils will have a major impact on the management of neutrophil diseases and may provide new information for comparative studies concerning pathological states.

## Results

### Isolation and characterization of human PMNs

**Figure 1:**
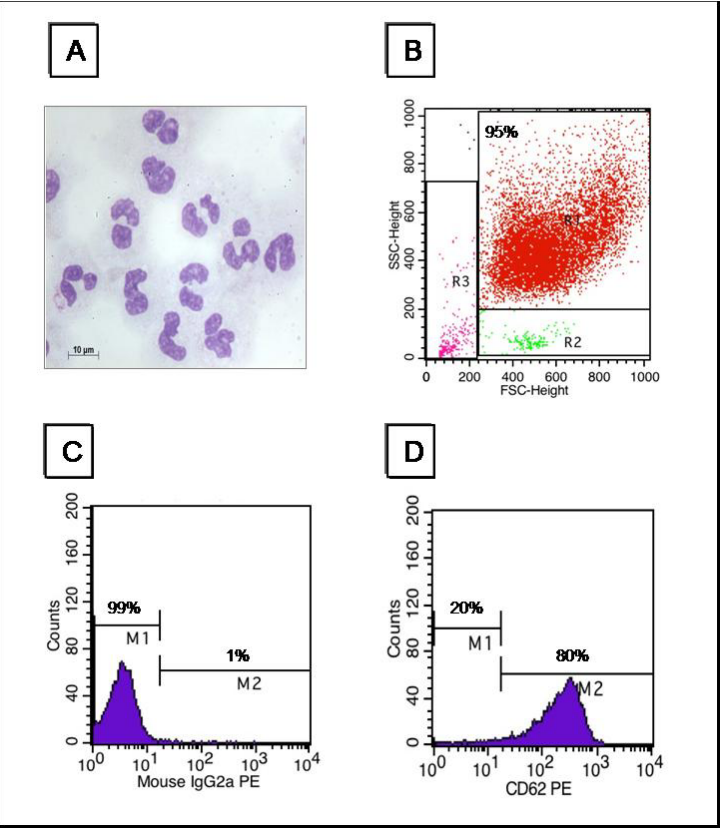
**Analysis of human neutrophil morphology, size and activation status**. A) Neutrophils stained with May-Grunwald-Giemsa were visualized by light microscopy at 1000 × magnification; B) homogeneity of the cells, 95.2% neutrophils (R1), 2.9% mononuclear cells (R2) and 1.9% RBCs and/or debris (R3), was evaluated by flow cytometry based on size (FSC) and internal complexity (SSC); C) analysis of the activation status using IgG2a (99%) as control isotype, and D) anti-CD62-L antibody indicating that 80% of the neutrophils were not activated.

The Percoll gradient method was used to isolate PMNs from human blood. An average of 2.5 ± 0.7 × 10^7 ^cells were obtained from 40 ml of human blood (Figure 1A), with a PMN homogeneity of 93 ± 2.8%. In order to eliminate residual contaminants, we introduced an additional step to lyses the contaminating red blood cells (RBCs) and 3 washes with Hanks' solution to eliminate contaminating monocytes/lymphocytes. Nevertheless, 95.2% of the PMNs (region R1 of Figure 1B) may still be contaminated by 2.9% of other leukocytes and 1.9% of RBCs and/or debris, as indicated by the presence of cells in regions R2 and R3 of Figure 1B. Another criterion used to characterize PMNs was the evaluation of the activation state of the cells. PMNs were labeled with the control isotype IgG2a and with CD62-L (or L-selectin), an adhesion molecule that play a role in the initial adhesion of neutrophils to endothelium. CD62-L is rapidly lost upon activation of phagocytic cells as a result of proteolytic cleavage, determining the initiation of the inflammatory response by the binding of lymphocytes to endothelium and the rolling of leucocytes at sites of tissue injury. Resting neutrophils express CD62-L and the presence of this glycoprotein indicates that neutrophils are not activated. As illustrated in Figures 1C and 1D, at least 80% of PMNs presented CD62-L on their surfaces and therefore were in the resting state. Of 25 cell preparations, 83.7 ± 11% showed CD62-L label. Cells of more than 95% homogeneity and 80% of resting cells were extracted and submitted to shotgun proteomic analysis.

### Proteome of resting human neutrophils

**Figure 2:**
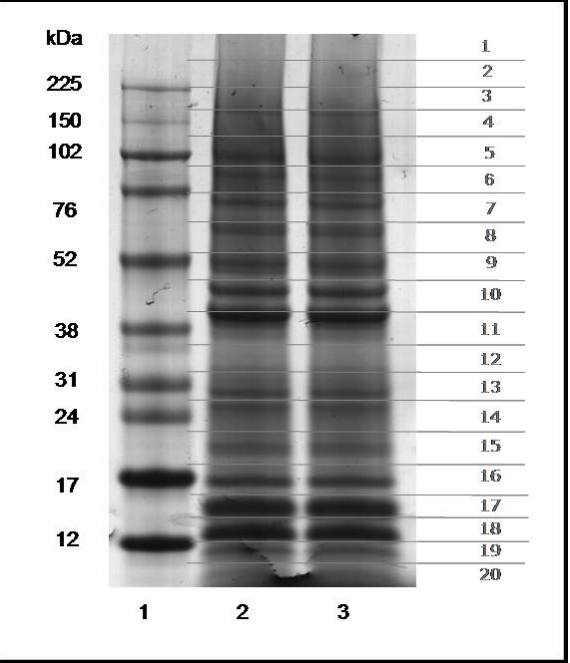
**Total extract of resting human neutrophils separated by 1D SDS-PAGE**. The protein extract was submitted to SDS-PAGE separation, stained with Coomassie Blue and cut into 20 horizontal bands. Each gel band was hydrolyzed with trypsin and the tryptic peptides were analyzed by LC-MS/MS. Lane 1: molecular weight standards (kDa); Lanes 2 and 3: 50 μg in duplicate of human neutrophil extract.

**Figure 3:**
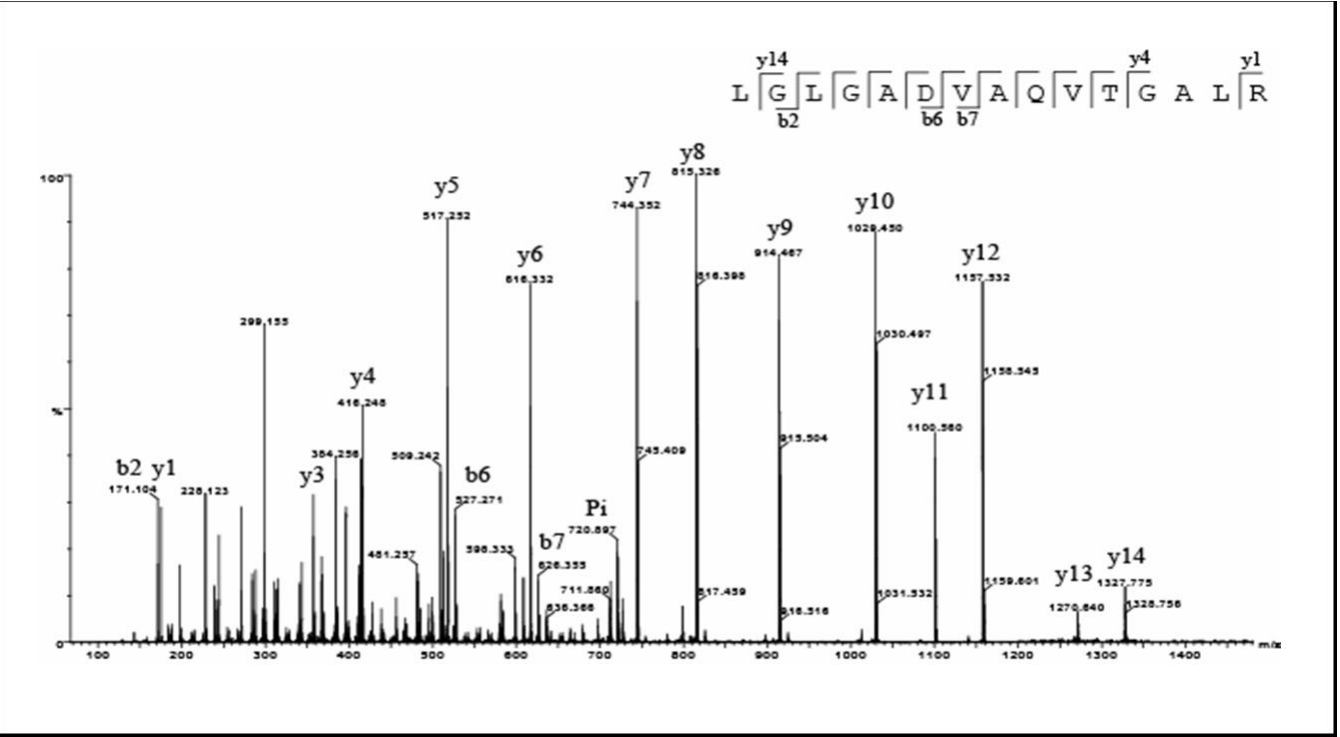
**MS/MS profile of ion M+2H = 720.887**. Tandem mass spectrum of parent ion (indicated by Pi in the Figure). As the result of collisions, the peptide was randomly fragmented at each peptide bond, resulting in carboxy-terminal y ions or amino-terminal b ions. When the fragment masses were submitted to Mascot, the peptide was identified as LGLGADVAQVTGALR from protein matrix metalloproteinase-9 (MMP-9) (inset, with detected y and b ions represented).

Isolated PMNs were disrupted with a neutral lysis buffer containing 1% SDS, 1% Triton X100, 50 mM TRIS, pH 7.5, 150 mM NaCl, and 10% (v/v) protease inhibitors. Fifty micrograms of the neutrophil extract was separated by 4-12% 1D-SDS-PAGE in duplicate, and each gel lane was cut into 20 pieces (Figure 2). The fragments were submitted to in-gel digestion and the resulting tryptic peptides were analyzed by HPLC (Ultimate3000) coupled to the Q-TOF Ultima Global mass spectrometer. The identification of proteins on the basis of at least 2 sequenced peptides and with a minimal score of 38 for each peptide (indicating p-values less than 0.05 per peptide according to Mascot) was immediately accepted. We accepted the validation of proteins identified on the basis of 1 peptide only when the score was at least twice the value for acceptance of MS/MS sequenced peptides. This corresponds to a Mascot Score of more than 76 which is equivalent to a false-positive rate of 5/1000. In addition, a peptide had to contain at least 7 amino acids and its sequence had to be confirmed by manual validation. Manual validation included the assignment of major peaks, the occurrence of uninterrupted y- or b-ion series of at least 3 consecutive amino acids. In order to help the reader to identify these confidence levels and to show that they are statistically acceptable, the sequenced peptides summarized in the additional file 1 were divided into 2 categories: 1) all proteins with at least two peptides each having a minimum score of 38 and 2) proteins with only one peptide but a Mascot Score higher than 76. Figure 3 shows an example of a MS/MS spectrum of a peptide of m/z 720.887. The Mascot engine identified this spectrum as the peptide LGLGADVAQVTGALR, present in the protein matrix metalloproteinase-9 precursor (MMP-9). Figure 3 also illustrates the fragmentation pattern and identification of y/b series on sequence (sequence input). In this example Mascot correlated 12 of the possible 14 y ions for this sequence, and 3 of 14 b ions, resulting in an identification score of 88 with 21% sequence coverage. The data for all peptides identified and the protein with which each peptide is associated are reported in Additional file 1. The table also contains information about protein mass, peptide length, observed charge, measured peptide mass (Da) and Mascot Score.

A total of 251 different proteins were identified in the present study. This is more than ten times the number of proteins identified by Castro et al (2006) [14] and almost five times the number of proteins identified by Piubelli et al (2002) [16] using a total extract of neutrophils. We also compared the proteins identified in the present study with those identified by subcellular proteome of human neutrophil granules [10], of secretory vesicles and plasma membrane [12,13] and of cytoskeleton [11]. Table 1 lists the number of proteins identified in the previous studies, as well as the subcellular fractionation used, the proteomic methodology employed and the number of proteins in common with the other studies. In Additional file 2 the proteins identified in the present study are listed in alphabetical order and their identification in other studies is indicated by a + symbol. The 55 proteins identified for the first time in human neutrophils are listed in Additional File 3.

**Table 1:** Comparison of the proteins identified in the present study and the proteins identified in other neutrophil proteome studies.

Proteome studies	Piubelli et al [16]	Lominadze et al [10]	Castro et al [14]	Jethwaney et al [12]	Uriarte et al [13]	Xu et al [11]	Present study
Extract or organelles	Total extract	Granules	Total extract	Secretory vesicles and plasma membranes	Secretory vesicles and plasma membranes	Cytoskeleton	Total extract
Methodology employed	2-DE	2-DE and LC-MS/MS	2-DE	2-DE and LC-MS/MS	LC-MS/MS	2-DE and LC-MS/MS	LC-MS/MS
Number of proteins identified	52	286	22	80	1118	138	251
Number of proteins in common with the present study	19 (36%)	74 (26%)	1 (4%)	32 (40%)	67 (6%)	34 (25%)	-

All of the studies were carried out on human neutrophils except for the one by Piubelli et al [16], who studied rat neutrophils

**Figure 4:**
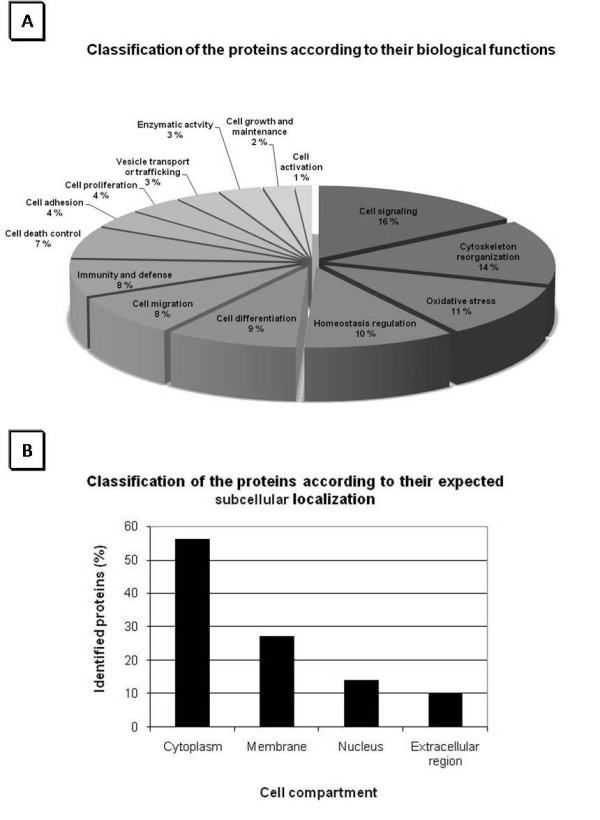
**Protein Ontology**. Identified proteins were classified according to their (A) biological functions and (B) expected subcellular localization.

Furthermore, proteins were analyzed with GeneTools. The analysis revealed a notable identification of proteins participating in cell signaling, cytoskeleton reorganization, oxidative stress and homeostasis regulation. The full classification of the proteins according to their biological functions is demonstrated in a pie chart in Figure 4. This figure also shows the classifications of the proteins identified here according to their expected subcellular localization, which revealed that almost 60% of the proteins are expected to be found in the cytoplasm, 27% are membrane proteins, 14% nuclear proteins, and 10% extracellular proteins.

## Discussion

Neutrophils are important inflammatory cells that are implicated in a wide range of diseases. Although there are few data available on proteomic analysis of human neutrophils [10] this is an area of interest growing that is likely to be significant in future investigations. Our group has recently demonstrated that the acute phase protein α-1 acid glycoprotein (AGP) is an inhibitor of neutrophil migration [17].

The global analysis of proteins has been enhanced by methodologies that provide higher sensitivity for protein identification [18]. The use of 2-DE to separate proteins has some limiting factors, since the total area of the gel limit the number of proteins that can be separated. Many of the spots detected in the gels are actually variations of the products of post-translationally modified proteins [19], that are not usually detected by shotgun sequencing and thus can be a limiting factor of the shotgun approach. Less abundant proteins and transmembrane proteins were not easily extracted or identified in 2-DE studies [20]. Since the publication of the proteome of rat polymorphonuclear leukocytes by Piubelli et al (2002) there have been studies concerning human neutrophils but most of them were subcellular proteomes.

Lominadze et al [10] determined the proteome of 3 different morphological types of human neutrophil granules using both 2-DE and LC-MS. The analysis of distribution of the 286 proteins in the granules revealed the presence of many differences among them and suggested functional heterogeneity, providing a basis for understanding the role of exocytosis in neutrophil biology. Jethwaney et al [12] and Uriarte et al [13] identified 74 and 1118 proteins, respectively, and compared the expression of proteins from secretory vesicles and plasma membrane of human neutrophils. Both studies identified differences between the two proteomes and contributed to the elucidation of the mechanisms and functional consequences of secretory vesicle exocytosis. Xu et al (2009) determined the subcellular proteome of the cytoskeleton of human neutrophils and among the 118 proteins identified they reported a series of cytoskeleton-associated proteins that had not been described. This was the broadest subcellular investigation to date of the neutrophil cytoskeletal proteome and the first proteomic analysis of any cell type of the phagosome skeleton [11]. These are different and complementary studies involving the same cell type and all are justified. The small number of proteins identical to those reported in other studies of neutrophils is probably due to the fact that most of the other studies focused on membrane proteins.

Even though such subcellular proteomics obtained relevant results, it is also important to characterize proteins from any organelle compartment easily accessible by a total cell extraction method. Using 2-DE/MS, Castro et al (2006) identified 22 proteins from resting human neutrophils that were potentially involved in the inflammatory response. This group described an initial proteomic analysis of human neutrophils which is the closest to our study [14]. However, actin-related protein 2 is the only protein in common between the proteomic study described by Castro et al. and the 251 proteins of the human resting neutrophil proteome described here.

Analysis of the protein ontology with GeneTools, a bioinformatics tool, revealed a notable identification of proteins participating in cell signaling, cytoskeleton reorganization, oxidative stress and homeostasis regulation. Such functional groups are in agreement for neutrophils biology in innate immunity, to allow them to kill pathogens and to orchestrate adaptive immune responses. The full classification of the proteins according to their biological functions is demonstrated on the pie chart in Figure 4. This figure also shows the classifications of the proteins identified here according to their expected subcellular localization. As expected, almost 60% of the proteins were found in the cytoplasm, while 27% were membrane proteins, 14% nuclear proteins and 10% extracellular proteins. We were able to identify 131 proteins that are potentially relevant to the cellular function of neutrophils not yet described in any of the papers describing neutrophils cited above. Fifty-five out of 131 are novel described for the first time in human neutrophils and are listed in the Additional File 3.

Centaurin beta-1 is another example of a protein described for the first time in human neutrophils. High CENTB1 expression in spleen, thymus, and bone marrow; intermediate expression in lung and testis; and low expression in prostate and ovary have been reported [21]. However, the expression in the intestine and immune cells was apparently not assessed. Yamamoto-Furusho et al (2006) detected the expression of CENTB1 in THP-1, Jurkat, and B cells under basal conditions. To our knowledge, the present study is the first to report this protein in human neutrophils. CENTB1 is a cytoplasmic protein that interacts with nucleotide-binding oligomerization domain -1 and -2 (NOD1 and NOD2) to down-regulate the NF-κB activity [22].

NF-κB is a key intracellular signaling molecule that controls the transcriptional activity of various promoters of pro-inflammatory cytokines, cell surface receptors, transcription factors, and adhesion molecules involved in inflammation [23]. In addition to modulating the transcription of immunomodulatory genes, NF-κB also plays an important role in programmed cell death (apoptosis), presumably by regulating the expression of genes important in regulating cell death [24]. In particular, decreased NF-κB activation results in increased apoptosis and decreased cell life spans. Based on these observations, the inhibitors of NF-κB have therapeutic potential for the treatment of in individuals with cancer, HIV-1 infection, or a wide variety of inflammatory diseases. The generation of highly specific and effective NF-κB inhibitors is of great interest [25].

Given the ability of neutrophils to synthesize several immunoregulatory proteins and to contribute to host defense processes, in addition to their traditional role as phagocytes, it has become evident that this cell type can modulate ongoing inflammatory or immune responses by the production of a wide range of cytokines and chemokines [26]. Since centaurin-beta-1 down-regulates the NF-κB activity via NODs pathways, it could be considered to be useful in the treatment of chronic inflammatory disorders in which neutrophils predominate, acting as a specific modulator of NF-κB functions.

## Conclusion

Since our main objective was to identify the largest number of proteins, the present report describes a catalog of human neutrophil proteins. Thanks to this inventory, we showed for the first time the expression of 55 proteins of human neutrophils, such as centaurin beta-1, a cytoplasmic protein that interacts with NOD-1 and -2 to down-regulate NF-κB activity. The precise understanding of the protein composition of neutrophils is supposed to contribute to further studies and may provide new information for comparative studies concerning pathological states. This would also have a major impact on drug discovery and development in order to prevent or manage neutrophil diseases.

## Methods

PMNs were isolated from healthy donors from the Protein Chemistry Center or of the Fundação Hemocentro of Ribeirão Preto, São Paulo. This study was approved by the Research Ethics Committee of the Federal University of São Paulo/São Paulo Hospital (# 1706/05).

### Neutrophil Isolation

Neutrophils (~4 × 10^7 ^cells) were isolated as described by Fraticelli et al. (1996). Briefly, blood from healthy donors was collected into tubes containing EDTA and applied to Percoll gradients (Amersham Biosciences) of the following concentrations: 72%, 63%, 54% and 45%. The gradient was centrifuged (30 min, 650 *g *and 25°C) and the granulocyte pellet was collected and washed in a buffer containing 150 mM NH_4_Cl, 10 mM NaHCO_3_, 1 mM EDTA for erythrocyte lysis and then in Hanks' solution. The cells were characterized by flow cytometry on the basis of their size, internal complexity and the expression of CD62L. PMNs were also characterized by light microscopy and were at least 95% homogeneous and 80% was not activated.

After isolation, 1 × 10^7^ neutrophils were suspended in 500 μL of a 1% SDS, 1% Triton X100, 50 mM TRIS, pH 7.5, 150 mM NaCl, and 10% (v/v) protease inhibitors (P8340, Sigma-Aldrich, MO) buffer to extract the proteins, which were quantified by the method of Bradford [27]. The neutrophil extract was stored in liquid nitrogen.

### SDS-PAGE

The total extract of human resting neutrophils was mixed with electrophoretic sample buffer (NuPAGE kit, Invitrogen, CA) containing 1 μL 100 mM DTT, and boiled for 5 min at 56°C prior to the electrophoretic run (duplicates of 50 μg each). Proteins were separated using a NuPage 4-12% Bis Tris Gel in MES (Invitrogen) at 200 V constant voltage for 30 min. The proteins were visualized using a Colloidal Coomassie Novex kit (Invitrogen). After staining, the gel lane containing the total neutrophil extract was cut into 20 fractions as described by de Souza et al [28], sliced into small pieces and submitted to digestion with trypsin in the gel.

### Protein Digestion

The proteins in the gel fragments were reduced with 10 mM DTT for 1 h at 56°C and alkylated with 55 mM iodoacetamide for 45 min at room temperature. The reduced and alkylated peptides were digested with 2% trypsin (w/w) (Sequence grade modified, Promega, WI, USA) for 16 h at 37°C in 50 mM NH_4_HCO_3_, pH 8.0. The reaction was stopped by acidification with 2% trifluoroacetic acid (Fluka,-Buchs, Germany). The resulting peptide mixture was desalted on RP-C18 STAGE tips [29] and diluted in 0.1% trifluoroacetic acid for nano-HPLC-MS analysis.

### Capillary LC-MS/MS Analysis

The tryptic peptide digests of the proteins were submitted to HPLC (Ultimate3000, LC Packings) coupled to the Q-TOF Ultima Global instrument (Waters, Micromass, MA) with 2.4 kV ionization. Peptides were separated on a C18 reverse phase column (0.075 × 150 mm) at 0.2 μL/min using a binary solvent system made up of Solvent A that was 2% acetonitrile (ACN) in 0.1% trifluoroacetic acid, whereas solvent B was aqueous 80% ACN in 0.1% trifluoroacetic acid. Peptides were eluted from the column with a linear gradient from 5-60% solvent B over 85 min, followed by 60-90% of solvent B over 10 min and finalizing with 90% solvent B for 5 min. Each full MS scan was carried out in the data-dependent mode, which was followed by MS/MS of the three most intense ions. To optimize peptide coverage, a mass/charge exclusion list was maintained so that the same peptide was not selected for MS/MS within a period of 40 seconds. The collision energy used for peptide fragmentation was varied according to the mass and charge of the ion. To confirm the reliability of the MS results, two preparations of total neutrophil extract were analyzed in duplicate.

### Database Searches

LC-MS/MS data were converted and the list of masses containing all the fragment information was submitted to Mascot (Matrix Science version 2.1) in order to identify the proteins using the International Protein Index (IPI) database for human proteins (IPI human, version 3.38), modified in-house to contain the reverse sequence of all entries to be used as a false-positive control, plus common contaminants such as trypsin and BSA. The search was performed using the following parameters: maximum of three missed trypsin cleavages, carbamidomethylation (Cys) as fixed variation, oxidation (Met) and acetylation (N-terminal of the protein) as variable modifications, and mass accuracy of 0.2 Da for both precursor ions and MS/MS data. Peptides with a minimum Mascot score of 38 indicate identifications with an error of less than 5% (p < 0.05). Proteins matching at least two peptides by Mascot were accepted automatically while identifications on the basis of only one peptide were accepted if the score was at least twice the threshold value for acceptance of MS/MS sequenced peptides (76) and using an MS/MS fragment of at least 7 amino acids, and after manual validation [30]. Spectra and protein validation were performed using an open source software called MSQuant, extensively used for LC-MS/MS data analysis [31] to control false-positive identifications [32]. With these parameters, we did not detect any false-positive identification using the reverse sequence entries in the database. This proteomic analysis was performed twice in duplicate with one neutrophil donor.

### Protein Ontology

The identified proteins were submitted to Genetools software [33] and their ontology was classified according to the biological processes in which they participate and to the expected subcellular localization.

## Competing interests

The authors declare that they have no competing interests.

## Authors' contributions

GGT contributed to the overall conception and design of the project and carried out the experiments, analysis and interpretation of the data, and preparation of the manuscript. IdS participated in the isolation of PMNs and contributed to data analysis and interpretation. HJL contributed with technical support. JCR and RC contributed with critical discussions of PMN biology and mass spectrometry, respectively. HGW contributed with proteomic data acquisition. GAdS contributed with proteomic data acquisition, proteomic interpretation and a critical review of the manuscript. LJG contributed with the conception and design of the project, manuscript preparation and critical review of the manuscript. All authors have read and approved the final manuscript.

## Supplementary Material

Additional file 1 **Human neutrophil tryptic peptides identified by Mascot score ≥ 38 (p < 0.05) of data obtained with a Q-TOF mass spectrometer**. The data concern to the tryptic peptides that were identified and contain information such as the access number, mass, sequence, length, charge and score of each peptide. Click here for file

Additional file 2 **Comparison of the proteins identified in the present study with the proteins identified in other studies of human neutrophil proteome**. The data summarize the proteins that were identified in the present study that were also identified by previous studies involving human neutrophils proteome. Click here for file

Additional file 3 **Proteins identified in the present study that are being described here for the first time in human neutrophils**. The data lists the proteins identified here that were described as human neutrophil proteins for the first time. Click here for file
