# Supplementary material for: Proteomic analysis of total cellular proteins of human neutrophils
Source: Proteome Sci. 2009 Aug 31;7:32. doi: 10.1186/1477-5956-7-32 (PMC3224919; doi:10.1186/1477-5956-7-32)
Supplement: Additional file 2 — Comparison of the proteins identified in the present study with the proteins identified in other studies of human neutrophil proteome. The data summarize the proteins that were identified in the present study that were also identified by previous studies involving human neutrophils proteome. [file 1477-5956-7-32-S2.doc]

### Additional file 2 – Comparison of the proteins identified in the present study with the proteins identified by other neutrophil proteome studies.

| **Number of proteins** | **Acess number** | **Proteins identified in the present study** | **Piubelli et al [23]**  **Total extract** | **Lominadze et al [17]**  **Granules** | **Castro et al [21**  **Total extrat** | **Jethwaney et al [19]**  **Secretory vesicles and plasma membranes** | **Uriarte et al [20]**  **Secretory vesicles and plasma membranes** | **Xu et al [18]**  **Cytoskeleton** |
| --- | --- | --- | --- | --- | --- | --- | --- | --- |
| 1 | IPI00021263 | 14-3-3 protein zeta/delta | + | + | ─ | + | ─ | + |
| 2 | IPI00792712 | 39 kDa protein | ─ | ─ | ─ | ─ | ─ | ─ |
| 3 | IPI00179330 | 40S ribosomal protein S27a | ─ | ─ | ─ | ─ | ─ | ─ |
| 4 | IPI00217143 | 57 kDa protein | ─ | ─ | ─ | ─ | ─ | ─ |
| 5 | IPI00219525 | 6-phosphogluconate dehydrogenase, decarboxylating | ─ | ─ | ─ | ─ | ─ | ─ |
| 6 | IPI00013698 | Acid ceramidase | ─ | ─ | ─ | ─ | ─ | ─ |
| 7 | IPI00025849 | Acidic leucine-rich nuclear phosphoprotein 32 family member A | ─ | ─ | ─ | ─ | ─ | ─ |
| 8 | IPI00021439 | Actin, cytoplasmic 1 | + | + | ─ | + | + | + |
| 9 | IPI00021440 | Actin, cytoplasmic 2 | + | ─ | ─ | ─ | + | + |
| 10 | IPI00005159 | Actin-related protein 2 | ─ | ─ | + | + | ─ | + |
| 11 | IPI00005160 | Actin-related protein 2/3 complex subunit 1B | ─ | ─ | ─ | ─ | + | ─ |
| 12 | IPI00005161 | Actin-related protein 2/3 complex subunit 2 | ─ | ─ | ─ | ─ | ─ | + |
| 13 | IPI00005162 | Actin-related protein 2/3 complex subunit 3 | ─ | ─ | ─ | ─ | ─ | + |
| 14 | IPI00554811 | Actin-related protein 2/3 complex subunit 4 | ─ | ─ | ─ | ─ | ─ | ─ |
| 15 | IPI00550234 | Actin-related protein 2/3 complex subunit 5, Isoform 1 | ─ | ─ | ─ | ─ | + | ─ |
| 16 | IPI00007280 | Actin-related protein 2/3 complex subunit 5, Isoform 2 | ─ | ─ | ─ | ─ | + | ─ |
| 17 | IPI00028091 | Actin-related protein 3 | ─ | + | ─ | ─ | + | ─ |
| 18 | IPI00218693 | Adenine phosphoribosyltransferase | ─ | ─ | ─ | ─ | ─ | ─ |
| 19 | IPI00012007 | Adenosylhomocysteinase | ─ | ─ | ─ | ─ | ─ | ─ |
| 20 | IPI00215901 | Adenylate kinase isoenzyme 2, mitochondrial, Isoform 1 | ─ | ─ | ─ | ─ | ─ | ─ |
| 21 | IPI00026833 | Adenylosuccinate synthetase isozyme 2 | ─ | ─ | ─ | ─ | ─ | ─ |
| 22 | IPI00008274 | Adenylyl cyclase-associated protein 1 | ─ | ─ | ─ | ─ | + | + |
| 23 | IPI00031131 | Adipocyte plasma membrane-associated protein | ─ | + | ─ | + | + | ─ |
| 24 | IPI00026240 | ADP-ribosyl cyclase 2 | ─ | ─ | ─ | + | ─ | ─ |
| 25 | IPI00215914 | ADP-ribosylation factor 1 | ─ | ─ | ─ | ─ | ─ | ─ |
| 26 | IPI00018871 | ADP-ribosylation factor-like protein 8B | ─ | ─ | ─ | ─ | ─ | ─ |
| 27 | IPI00022434 | Albumin | ─ | + | ─ | + | + | ─ |
| 28 | IPI00220271 | Alcohol dehydrogenase | ─ | ─ | ─ | ─ | ─ | ─ |
| 29 | IPI00553177 | Alpha-1-antitrypsin, Isoform 1 | ─ | ─ | ─ | + | ─ | ─ |
| 30 | IPI00013508 | Alpha-actinin-1 | ─ | + | ─ | + | + | + |
| 31 | IPI00013808 | Alpha-actinin-4 | ─ | ─ | ─ | + | + | ─ |
| 32 | IPI00465248 | Alpha-enolase, Isoform alpha-enolase | + | + | ─ | ─ | + | + |
| 33 | IPI00642211 | Aminopeptidase B | ─ | ─ | ─ | ─ | ─ | ─ |
| 34 | IPI00221224 | Aminopeptidase N | ─ | + | ─ | ─ | + | ─ |
| 35 | IPI00218918 | Annexin A1 | + | + | ─ | ─ | + | + |
| 36 | IPI00414320 | Annexin A11 | ─ | ─ | ─ | ─ | + | ─ |
| 37 | IPI00024095 | Annexin A3 | + | + | ─ | ─ | ─ | + |
| 38 | IPI00329801 | Annexin A5 | ─ | ─ | ─ | ─ | ─ | ─ |
| 39 | IPI00221226 | Annexin A6 | ─ | ─ | ─ | ─ | ─ | + |
| 40 | IPI00793199 | Annexin IV | ─ | ─ | ─ | ─ | ─ | ─ |
| 41 | IPI00002459 | Annexin VI isoform 2 | ─ | ─ | ─ | ─ | ─ | ─ |
| 42 | IPI00292532 | Antibacterial protein FALL-39 | ─ | ─ | ─ | ─ | ─ | ─ |
| 43 | IPI00001699 | Apoptosis-associated speck-like protein containing a CA, Isoform 1 | ─ | ─ | ─ | ─ | ─ | ─ |
| 44 | IPI00291560 | Arginase-1, Isoform 1 | ─ | ─ | ─ | ─ | ─ | ─ |
| 45 | IPI00018206 | Aspartate aminotransferase, mitochondrial | ─ | ─ | ─ | ─ | ─ | ─ |
| 46 | IPI00514381 | Associated transcript 1 | ─ | ─ | ─ | ─ | ─ | ─ |
| 47 | IPI00440493 | ATP synthase subunit alpha, mitochondrial | ─ | + | ─ | + | ─ | ─ |
| 48 | IPI00303476 | ATP synthase subunit beta, mitochondrial | + | + | ─ | + | + | + |
| 49 | IPI00021290 | ATP-citrate synthase | ─ | ─ | ─ | ─ | ─ | ─ |
| 50 | IPI00827847 | Bactericidal permeability-increasing protein | ─ | + | ─ | ─ | + | ─ |
| 51 | IPI00027851 | Beta-hexosaminidase alpha chain | ─ | + | ─ | ─ | ─ | ─ |
| 52 | IPI00305010 | Calcineurin-like phosphoesterase domain-containing protein 1 | ─ | ─ | ─ | ─ | ─ | ─ |
| 53 | IPI00032561 | Calcium-binding protein 39 | ─ | ─ | ─ | ─ | ─ | ─ |
| 54 | IPI00075248 | Calmodulin | + | ─ | ─ | ─ | ─ | ─ |
| 55 | IPI00027252 | Calponin-2 | ─ | ─ | ─ | ─ | ─ | ─ |
| 56 | IPI00020599 | Calreticulin | + | + | ─ | ─ | + | ─ |
| 57 | IPI00218782 | Capping protein | ─ | ─ | ─ | ─ | ─ | ─ |
| 58 | IPI00027412 | Carcinoembryonic antigen-related cell adhesion molecule 6 | ─ | ─ | ─ | ─ | + | ─ |
| 59 | IPI00013972 | Carcinoembryonic antigen-related cell adhesion molecule 8 | ─ | ─ | ─ | ─ | + | ─ |
| 60 | IPI00009938 | Carcinoembryonic antigen-related cell adhesion molecule, Isoform A | ─ | + | ─ | ─ | + | ─ |
| 61 | IPI00465436 | Catalase | ─ | + | ─ | ─ | + | + |
| 62 | IPI00028064 | Cathepsin G | ─ | + | ─ | ─ | + | ─ |
| 63 | IPI00215998 | CD63 antigen | ─ | ─ | ─ | ─ | ─ | ─ |
| 64 | IPI00017672 | cDNA FLJ25678 fis, clone TST04067, highly similar to PURINE NUCLEOSIDE P | ─ | ─ | ─ | ─ | ─ | ─ |
| 65 | IPI00014199 | Centaurin-beta-1 | ─ | ─ | ─ | ─ | ─ | ─ |
| 66 | IPI00002147 | Chitinase-3-like protein 1 | ─ | ─ | ─ | ─ | ─ | ─ |
| 67 | IPI00010896 | Chloride intracellular channel protein 1 | ─ | ─ | ─ | + | ─ | ─ |
| 68 | IPI00012011 | Cofilin-1 | + | + | ─ | ─ | ─ | + |
| 69 | IPI00010133 | Coronin-1A | ─ | + | ─ | + | ─ | + |
| 70 | IPI00867509 | Coronin-1C_i3 protein | ─ | ─ | ─ | ─ | ─ | ─ |
| 71 | IPI00032325 | Cystatin-A | ─ | ─ | ─ | ─ | ─ | ─ |
| 72 | IPI00218646 | Cytochrome b-245 heavy chain | ─ | + | ─ | + | ─ | ─ |
| 73 | IPI00293867 | D-dopachrome decarboxylase | ─ | ─ | ─ | ─ | ─ | ─ |
| 74 | IPI00015911 | Dihydrolipoyl dehydrogenase, mitochondrial | ─ | ─ | ─ | ─ | ─ | ─ |
| 75 | IPI00215911 | DNA-(apurinic or apyrimidinic site) lyase | ─ | ─ | ─ | ─ | ─ | ─ |
| 76 | IPI00060181 | EF-hand domain-containing protein 2 | ─ | + | ─ | ─ | ─ | + |
| 77 | IPI00396485 | Elongation factor 1-alpha1 | ─ | ─ | ─ | ─ | ─ | ─ |
| 78 | IPI00023048 | Elongation factor 1-delta | ─ | ─ | ─ | ─ | ─ | ─ |
| 79 | IPI00186290 | Elongation factor 2 | ─ | ─ | ─ | ─ | ─ | ─ |
| 80 | IPI00219682 | Erythrocyte band 7 integral membrane protein | ─ | ─ | ─ | + | ─ | ─ |
| 81 | IPI00005969 | F-actin-capping protein subunit alpha-1 | ─ | + | ─ | ─ | ─ | + |
| 82 | IPI00026182 | F-actin-capping protein subunit alpha-2 | ─ | + | ─ | ─ | + | + |
| 83 | IPI00007797 | Fatty acid-binding protein, epidermal | ─ | ─ | ─ | ─ | ─ | ─ |
| 84 | IPI00216699 | Fermitin family homolog 3 | ─ | ─ | ─ | ─ | ─ | ─ |
| 85 | IPI00738499 | Ferritin light chain | ─ | + | ─ | ─ | ─ | ─ |
| 86 | IPI00012555 | Ficolin-1 | ─ | + | ─ | ─ | + | + |
| 87 | IPI00302592 | filamin A, alpha isoform 1 | ─ | + | ─ | ─ | + | + |
| 88 | IPI00873810 | FK506-binding protein 1A | ─ | ─ | ─ | ─ | ─ | ─ |
| 89 | IPI00465439 | Fructose-bisphosphate aldolase A | ─ | ─ | ─ | ─ | + | + |
| 90 | IPI00418262 | Fructose-bisphosphate aldolase C | ─ | ─ | ─ | ─ | ─ | ─ |
| 91 | IPI00465431 | Galectin-3 | ─ | ─ | ─ | ─ | + | ─ |
| 92 | IPI00465121 | Galphai2 protein | ─ | ─ | ─ | ─ | ─ | ─ |
| 93 | IPI00026314 | Gelsolin, Isoform 1 | ─ | + | ─ | ─ | + | + |
| 94 | IPI00646773 | Gelsolin, Isoform 2 | ─ | + | ─ | ─ | + | + |
| 95 | IPI00848090 | gelsolin-like capping protein | ─ | + | ─ | ─ | + | + |
| 96 | IPI00216008 | Glucose-6-phosphate 1-dehydrogenase, Isoform Long | ─ | ─ | ─ | ─ | ─ | ─ |
| 97 | IPI00027497 | Glucose-6-phosphate isomerase | ─ | + | ─ | ─ | ─ | ─ |
| 98 | IPI00003919 | Glutaminyl-peptide cyclotransferase | ─ | + | ─ | ─ | ─ | ─ |
| 99 | IPI00219025 | Glutaredoxin-1 | ─ | ─ | ─ | ─ | ─ | ─ |
| 100 | IPI00016862 | Glutathione reductase, Isoform Mitochondrial | ─ | ─ | ─ | ─ | ─ | ─ |
| 101 | IPI00219757 | Glutathione S-transferase P | ─ | ─ | ─ | ─ | ─ | ─ |
| 102 | IPI00010706 | Glutathione synthetase | ─ | ─ | ─ | ─ | ─ | ─ |
| 103 | IPI00019755 | Glutathione transferase omega-1 | ─ | ─ | ─ | ─ | ─ | ─ |
| 104 | IPI00219018 | Glyceraldehyde-3-phosphate dehydrogenase | + | ─ | ─ | ─ | + | ─ |
| 105 | IPI00783313 | Glycogen phosphorylase, liver form | ─ | ─ | ─ | ─ | + | ─ |
| 106 | IPI00007067 | Golgi-associated plant pathogenesis-related protein 1 | ─ | + | ─ | + | ─ | ─ |
| 107 | IPI00004524 | Grancalcin | ─ | + | ─ | ─ | ─ | + |
| 108 | IPI00290928 | Guanine nucleotide-binding protein alpha-13 subunit | ─ | ─ | ─ | ─ | ─ | ─ |
| 109 | IPI00026268 | Guanine nucleotide-binding protein G(I)/G(S)/G(T) subunit beta-1 | ─ | ─ | ─ | + | ─ | ─ |
| 110 | IPI00641737 | Haptoglobin | ─ | + | ─ | ─ | + | ─ |
| 111 | IPI00003865 | Heat shock cognate 71 kDa protein, Isoform 1 | ─ | ─ | ─ | ─ | ─ | + |
| 112 | IPI00003799 | Heme-binding protein 2, Isoform 2 | ─ | ─ | ─ | ─ | ─ | ─ |
| 113 | IPI00654755 | Hemoglobin subunit beta | + | + | ─ | ─ | + | ─ |
| 114 | IPI00759596 | Heterogeneous nuclear ribonucleoproteins C1/C2, Isoform 4 | ─ | ─ | ─ | ─ | ─ | ─ |
| 115 | IPI00059366 | histone family, member Y isoform 2 | ─ | ─ | ─ | ─ | ─ | ─ |
| 116 | IPI00003935 | Histone H2B type 2-E | ─ | + | ─ | ─ | ─ | ─ |
| 117 | IPI00453473 | Histone H4 | ─ | + | ─ | ─ | + | ─ |
| 118 | IPI00431645 | HP protein | ─ | ─ | ─ | ─ | ─ | ─ |
| 119 | IPI00847536 | HSPA1B;HSPA1A Heat shock 70 kDa protein 1 | ─ | + | ─ | ─ | + | ─ |
| 120 | IPI00003362 | HSPA5 protein | ─ | + | ─ | ─ | + | ─ |
| 121 | IPI00218493 | Hypoxanthine-guanine phosphoribosyltransferase | ─ | ─ | ─ | ─ | ─ | ─ |
| 122 | IPI00001639 | Importin subunit beta-1 | ─ | ─ | ─ | ─ | ─ | ─ |
| 123 | IPI00217987 | Integrin alpha-M | ─ | + | ─ | + | + | + |
| 124 | IPI00291792 | Integrin beta-2 | ─ | + | ─ | + | + | ─ |
| 125 | IPI00031620 | Intercellular adhesion molecule 3 | ─ | + | ─ | + | + | ─ |
| 126 | IPI00005198 | Interleukin enhancer-binding factor 2 | ─ | ─ | ─ | ─ | + | ─ |
| 127 | IPI00000045 | Interleukin-1 receptor antagonist protein, Isoform 1 | ─ | ─ | ─ | ─ | ─ | ─ |
| 128 | IPI00304082 | Isochorismatase domain-containing protein 1 | ─ | ─ | ─ | ─ | ─ | ─ |
| 129 | IPI00027223 | Isocitrate dehydrogenase [NADP] cytoplasmic | ─ | ─ | ─ | ─ | ─ | ─ |
| 130 | IPI00011107 | Isocitrate dehydrogenase [NADP], mitochondrial | ─ | + | ─ | + | ─ | ─ |
| 131 | IPI00298860 | Lactoransferrin | ─ | + | ─ | + | ─ | ─ |
| 132 | IPI00022417 | Leucine-rich alpha-2-glycoprotein | ─ | ─ | ─ | ─ | ─ | ─ |
| 133 | IPI00219077 | Leukotriene A-4 hydrolase, Isoform 1 | ─ | ─ | ─ | ─ | ─ | + |
| 134 | IPI00217966 | L-lactate dehydrogenase A chain, Isoform 1 | ─ | ─ | ─ | ─ | ─ | ─ |
| 135 | IPI00219217 | L-lactate dehydrogenase B chain | ─ | ─ | ─ | ─ | ─ | ─ |
| 136 | IPI00012989 | Lysosomal alpha-mannosidase | ─ | + | ─ | ─ | ─ | ─ |
| 137 | IPI00019038 | Lysozyme C | ─ | + | ─ | ─ | + | ─ |
| 138 | IPI00293276 | Macrophage migration inhibitory factor | ─ | ─ | ─ | ─ | ─ | ─ |
| 139 | IPI00027341 | Macrophage-capping protein | ─ | ─ | ─ | ─ | ─ | ─ |
| 140 | IPI00291005 | Malate dehydrogenase, cytoplasmic | ─ | ─ | ─ | ─ | ─ | ─ |
| 141 | IPI00291006 | Malate dehydrogenase, mitochondrial | ─ | ─ | ─ | + | ─ | ─ |
| 142 | IPI00220143 | Maltase-glucoamylase,intestinal | ─ | + | ─ | ─ | + | ─ |
| 143 | IPI00027509 | Matrix metalloproteinase-9 | ─ | ─ | ─ | + | + | ─ |
| 144 | IPI00787992 | MHC class I antigen (Fragment) | ─ | + | ─ | + | ─ | ─ |
| 145 | IPI00872814 | Moesin | ─ | + | ─ | + | + | + |
| 146 | IPI00027409 | Myeloblastin | ─ | + | ─ | + | + | ─ |
| 147 | IPI00013163 | Myeloid cell nuclear differentiation antigen | ─ | ─ | ─ | ─ | + | ─ |
| 148 | IPI00236554 | Myeloperoxidase, Isoform H14 | ─ | + | ─ | + | + | ─ |
| 149 | IPI00007244 | Myeloperoxidase, Isoform H17 | ─ | + | ─ | + | + | ─ |
| 150 | IPI00335168 | Myosin light polypeptide 6, Non-muscle isoform | + | + | ─ | + | ─ | + |
| 151 | IPI00019502 | Myosin-9 | ─ | + | ─ | + | + | + |
| 152 | IPI00296526 | N-acetylglucosamine kinase | ─ | ─ | ─ | ─ | ─ | + |
| 153 | IPI00012102 | N-acetylglucosamine-6-sulfatase | ─ | ─ | ─ | ─ | ─ | ─ |
| 154 | IPI00027846 | Neutrophil collagenase (MMP8) | ─ | + | ─ | ─ | + | ─ |
| 155 | IPI00014338 | Neutrophil cytosol factor 4, Isoform 1 | ─ | ─ | ─ | ─ | ─ | ─ |
| 156 | IPI00299547 | Neutrophil gelatinase-associated lipocalin | ─ | ─ | ─ | ─ | ─ | ─ |
| 157 | IPI00021983 | Nicastrin, Isoform 1 | ─ | ─ | ─ | ─ | ─ | ─ |
| 158 | IPI00880164 | Nicotinate phosphoribosyltransferase, Isoform 3 | ─ | ─ | ─ | ─ | ─ | ─ |
| 159 | IPI00412498 | Nicotinate phosphoribosyltransferase, Isoform 4 | ─ | ─ | ─ | ─ | ─ | ─ |
| 160 | IPI00549467 | Nit protein 2 | ─ | ─ | ─ | ─ | ─ | ─ |
| 161 | IPI00019449 | Non-secretory ribonuclease | ─ | ─ | ─ | ─ | ─ | ─ |
| 162 | IPI00026260 | Nucleoside diphosphate kinase B | ─ | ─ | ─ | ─ | ─ | ─ |
| 163 | IPI00419585 | Peptidyl-prolyl cis-trans isomerase A | ─ | ─ | ─ | ─ | ─ | ─ |
| 164 | IPI00646304 | Peptidylprolyl isomerase B | ─ | ─ | ─ | ─ | ─ | ─ |
| 165 | IPI00024915 | Peroxiredoxin-5, Isoform Mitochondrial | ─ | ─ | ─ | ─ | ─ | ─ |
| 166 | IPI00220301 | Peroxiredoxin-6 | ─ | ─ | ─ | ─ | ─ | ─ |
| 167 | IPI00169383 | Phosphoglycerate kinase 1 | ─ | ─ | ─ | ─ | + | ─ |
| 168 | IPI00010471 | Plastin-2 | ─ | + | ─ | ─ | + | + |
| 169 | IPI00026546 | Platelet-activating factor acetylhydrolase IB subunit beta | ─ | ─ | ─ | ─ | ─ | ─ |
| 170 | IPI00216691 | Profilin-1 | + | + | ─ | ─ | + | + |
| 171 | IPI00017334 | Prohibitin | ─ | + | ─ | ─ | + | + |
| 172 | IPI00291922 | Proteasome subunit alpha type-5 | ─ | ─ | ─ | ─ | ─ | ─ |
| 173 | IPI00024175 | Proteasome subunit alpha type-7, Isoform 1 | ─ | ─ | ─ | ─ | ─ | + |
| 174 | IPI00000783 | Proteasome subunit beta type-8, Isoform 1 | ─ | ─ | ─ | ─ | ─ | ─ |
| 175 | IPI00000787 | Proteasome subunit beta type-9, Isoform LMP2.L | ─ | ─ | ─ | ─ | + | + |
| 176 | IPI00025252 | Protein disulfide-isomerase A3 | + | + | ─ | ─ | + | + |
| 177 | IPI00299571 | Protein disulfide-isomerase A6, Isoform 2 | ─ | ─ | ─ | ─ | + | ─ |
| 178 | IPI00329236 | Protein kinase C delta type | ─ | ─ | ─ | ─ | ─ | ─ |
| 179 | IPI00032313 | Protein S100-A4 | ─ | ─ | ─ | ─ | + | ─ |
| 180 | IPI00027463 | Protein S100-A6 | ─ | ─ | ─ | ─ | ─ | ─ |
| 181 | IPI00007047 | Protein S100-A8 | ─ | ─ | ─ | ─ | + | + |
| 182 | IPI00027462 | Protein S100-A9 | ─ | + | ─ | ─ | + | + |
| 183 | IPI00017526 | Protein S100-P | ─ | ─ | ─ | ─ | ─ | ─ |
| 184 | IPI00645319 | Protein tyrosine phosphatase, receptor type, C isoform 3 | ─ | + | ─ | ─ | + | ─ |
| 185 | IPI00026216 | Puromycin-sensitive aminopeptidase | ─ | ─ | ─ | ─ | ─ | ─ |
| 186 | IPI00219677 | Putative deoxyribose-phosphate aldolase | ─ | ─ | ─ | ─ | ─ | ─ |
| 187 | IPI00027007 | Putative neutrophil cytosol factor 1C | ─ | ─ | ─ | ─ | ─ | ─ |
| 188 | IPI00016255 | Putative phospholipase B-like1 | ─ | ─ | ─ | ─ | ─ | ─ |
| 189 | IPI00010402 | Putative uncharacterized protein | ─ | ─ | ─ | ─ | ─ | ─ |
| 190 | IPI00479186 | Pyruvate kinase isozymes M1/M2, Isoform M1 | ─ | ─ | ─ | ─ | ─ | + |
| 191 | IPI00031461 | Rab GDP dissociation inhibitor beta | ─ | + | ─ | ─ | ─ | ─ |
| 192 | IPI00009342 | Ras GTPase-activating-like protein IQGAP1 | ─ | ─ | ─ | ─ | ─ | ─ |
| 193 | IPI00016381 | Ras-related protein Rab-27A, Isoform Long | ─ | + | ─ | ─ | ─ | ─ |
| 194 | IPI00010270 | Ras-related C3 botulinum toxin substrate 2 | ─ | ─ | ─ | ─ | ─ | ─ |
| 195 | IPI00016513 | Ras-related protein Rab-10 | ─ | + | ─ | ─ | ─ | ─ |
| 196 | IPI00020436 | Ras-related protein Rab-11B | ─ | + | ─ | ─ | ─ | ─ |
| 197 | IPI00291928 | Ras-related protein Rab-14 | ─ | ─ | ─ | ─ | ─ | ─ |
| 198 | IPI00008964 | Ras-related protein Rab-1B | ─ | ─ | ─ | ─ | ─ | ─ |
| 199 | IPI00031169 | Ras-related protein Rab-2A | ─ | ─ | ─ | ─ | ─ | ─ |
| 200 | IPI00014376 | Ras-related protein Rab-31 | ─ | ─ | ─ | ─ | ─ | ─ |
| 201 | IPI00032808 | Ras-related protein Rab-3D | ─ | ─ | ─ | ─ | ─ | ─ |
| 202 | IPI00016339 | Ras-related protein Rab-5C | ─ | + | ─ | ─ | ─ | ─ |
| 203 | IPI00016342 | Ras-related protein Rab-7a | ─ | + | ─ | ─ | ─ | ─ |
| 204 | IPI00028481 | Ras-related protein Rab-8A | ─ | ─ | ─ | ─ | ─ | ─ |
| 205 | IPI00015148 | Ras-related protein Rap-1b | ─ | + | ─ | + | ─ | ─ |
| 206 | IPI00290328 | Receptor-type tyrosine-protein phosphatase eta | ─ | ─ | ─ | ─ | ─ | ─ |
| 207 | IPI00006988 | Resistin | ─ | ─ | ─ | ─ | ─ | ─ |
| 208 | IPI00003815 | Rho GDP-dissociation inhibitor 1 | ─ | ─ | ─ | ─ | ─ | ─ |
| 209 | IPI00003817 | Rho GDP-dissociation inhibitor 2 | + | ─ | ─ | ─ | + | ─ |
| 210 | IPI00020567 | Rho GTPase-activating protein 1 | ─ | ─ | ─ | ─ | ─ | ─ |
| 211 | IPI00017342 | Rho-related GTP-binding protein RhoG | ─ | ─ | ─ | ─ | ─ | ─ |
| 212 | IPI00550069 | Ribonuclease inhibitor | ─ | ─ | ─ | ─ | ─ | ─ |
| 213 | IPI00414896 | Ribonuclease T2, Isoform 1 | ─ | ─ | ─ | ─ | ─ | ─ |
| 214 | IPI00028635 | Ribophorin II | ─ | + | ─ | ─ | ─ | ─ |
| 215 | IPI00026513 | Ribose-5-phosphate isomerase | ─ | ─ | ─ | ─ | ─ | ─ |
| 216 | IPI00027444 | SERPIN B1 Leukocyte elastase inhibitor | ─ | ─ | ─ | ─ | ─ | ─ |
| 217 | IPI00010304 | Serpin B10 | ─ | ─ | ─ | ─ | ─ | ─ |
| 218 | IPI00022204 | Serpin B3 | ─ | ─ | ─ | ─ | ─ | ─ |
| 219 | IPI00025318 | SH3 domain-binding glutamic acid-rich-like protein | ─ | ─ | ─ | ─ | ─ | ─ |
| 220 | IPI00788068 | Similar to Arachidonate 5-lipoxygenase | ─ | ─ | ─ | ─ | ─ | ─ |
| 221 | IPI00003909 | Solute carrier family 2, facilitated glucose transporter member 3 | ─ | ─ | ─ | + | + | ─ |
| 222 | IPI00007765 | Stress-70 protein, mitochondrial precursor | ─ | ─ | ─ | ─ | ─ | ─ |
| 223 | IPI00022314 | Superoxide dismutase [Mn],mitochondrial | ─ | ─ | ─ | ─ | + | ─ |
| 224 | IPI00156689 | Synaptic vesicle membrane protein VAT-1 homolog | ─ | + | ─ | ─ | ─ | ─ |
| 225 | IPI00298994 | Talin-1 | ─ | + | ─ | ─ | ─ | ─ |
| 226 | IPI00216298 | Thioredoxin | ─ | ─ | ─ | ─ | ─ | ─ |
| 227 | IPI00024919 | Thioredoxin-dependent peroxide reductase, mitochondrial | ─ | ─ | ─ | ─ | ─ | ─ |
| 228 | IPI00292858 | Thymidine phosphorylase | ─ | ─ | ─ | ─ | ─ | ─ |
| 229 | IPI00413293 | Torsin-1A, Isoform 1 | ─ | ─ | ─ | ─ | ─ | ─ |
| 230 | IPI00744692 | Transaldolase | ─ | ─ | ─ | ─ | ─ | ─ |
| 231 | IPI00478231 | Transforming protein RhoA | ─ | ─ | ─ | ─ | ─ | ─ |
| 232 | IPI00550363 | Transgelin-2 | + | + | ─ | ─ | ─ | ─ |
| 233 | IPI00643920 | Transketolase | ─ | + | ─ | ─ | ─ | ─ |
| 234 | IPI00788802 | Transketolase variant (Fragment) | ─ | ─ | ─ | ─ | ─ | ─ |
| 235 | IPI00018768 | Translin | ─ | ─ | ─ | ─ | ─ | ─ |
| 236 | IPI00465028 | Triosephosphate isomerase, Isoform 1 | ─ | ─ | ─ | ─ | ─ | ─ |
| 237 | IPI00451401 | Triosephosphate isomerase, Isoform 2 | ─ | ─ | ─ | ─ | ─ | ─ |
| 238 | IPI00219526 | Tripeptidyl-peptidase 1, Isoform 1 | ─ | ─ | ─ | ─ | ─ | ─ |
| 239 | IPI00014581 | Tropomyosin alpha-1 chain, Isoform 1 | ─ | ─ | ─ | ─ | ─ | ─ |
| 240 | IPI00166768 | Tubulin alpha-1C chain | + | + | ─ | ─ | + | ─ |
| 241 | IPI00550917 | Twinfilin-2 | ─ | ─ | ─ | ─ | ─ | ─ |
| 242 | IPI00003949 | Ubiquitin-conjugating enzyme E2 N | + | ─ | ─ | ─ | ─ | ─ |
| 243 | IPI00645078 | Ubiquitin-like modifier-activating enzyme 1 | ─ | ─ | ─ | ─ | ─ | ─ |
| 244 | IPI00453476 | Uncharacterized protein ENSP00000348237 | ─ | ─ | ─ | ─ | ─ | ─ |
| 245 | IPI00016670 | UPF0404 protein C11orf59 | ─ | ─ | ─ | ─ | ─ | ─ |
| 246 | IPI00007812 | Vacuolar ATP synthase subunit B, brain isoform | ─ | ─ | ─ | ─ | ─ | ─ |
| 247 | IPI00030872 | Vascular non-inflammatory molecule 2 | ─ | ─ | ─ | ─ | ─ | ─ |
| 248 | IPI00301058 | Vasodilator-stimulated phosphoprotein | ─ | ─ | ─ | ─ | + | ─ |
| 249 | IPI00291175 | Vinculin, Isoform 1 | ─ | ─ | ─ | ─ | ─ | + |
| 250 | IPI00024145 | Voltage-dependent anion-selective channel protein 2 | ─ | ─ | ─ | ─ | ─ | ─ |
| 251 | IPI00216256 | WD repeat-containing protein 1, Isoform 2 | ─ | ─ | ─ | ─ | ─ | ─ |
