# Supplementary material for: Proteomic analysis of total cellular proteins of human neutrophils
Source: Proteome Sci. 2009 Aug 31;7:32. doi: 10.1186/1477-5956-7-32 (PMC3224919; doi:10.1186/1477-5956-7-32)
Supplement: Additional file 3 — Proteins identified in the present study that are being described here for the first time in human neutrophils. The data lists the proteins identified here that were described as human neutrophil proteins for the first time. [file 1477-5956-7-32-S3.docx]

### Additional file 3 - Proteins identified in the present study that are being described here for the first time in human neutrophils.

| **Number of proteins** | **Acess number** | **First time identified proteins in human neutrophils** |
| --- | --- | --- |
| 1 | IPI00179330 | 40S ribosomal protein S27a |
| 2 | IPI00013698 | Acid ceramidase |
| 3 | IPI00025849 | Acidic leucine-rich nuclear phosphoprotein 32 family member A |
| 5 | IPI00018871 | ADP-ribosylation factor-like protein 8B |
| 6 | IPI00001699 | Apoptosis-associated speck-like protein containing a CA, Isoform 1 |
| 7 | IPI00021290 | ATP-citrate synthase |
| 8 | IPI00305010 | Calcineurin-like phosphoesterase domain-containing protein 1 |
| 9 | IPI00032561 | Calcium-binding protein 39 |
| 10 | IPI00027252 | Calponin-2 |
| 11 | IPI00017672 | cDNA FLJ25678 fis, clone TST04067, highly similar to PURINE NUCLEOSIDE P |
| 12 | IPI00014199 | Centaurin-beta-1 |
| 13 | IPI00002147 | Chitinase-3-like protein 1 |
| 14 | IPI00293867 | D-dopachrome decarboxylase |
| 15 | IPI00015911 | Dihydrolipoyl dehydrogenase, mitochondrial |
| 16 | IPI00215911 | DNA-(apurinic or apyrimidinic site) lyase |
| 17 | IPI00023048 | Elongation factor 1-delta |
| 18 | IPI00186290 | Elongation factor 2 |
| 19 | IPI00019755 | Glutathione transferase omega-1 |
| 20 | IPI00290928 | Guanine nucleotide-binding protein alpha-13 subunit |
| 21 | IPI00759596 | Heterogeneous nuclear ribonucleoproteins C1/C2, Isoform 4 |
| 22 | IPI00001639 | Importin subunit beta-1 |
| 23 | IPI00304082 | Isochorismatase domain-containing protein 1 |
| 24 | IPI00219217 | L-lactate dehydrogenase B chain |
| 25 | IPI00027341 | Macrophage-capping protein |
| 26 | IPI00021983 | Nicastrin, Isoform 1 |
| 27 | IPI00880164 | Nicotinate phosphoribosyltransferase, Isoform 3 |
| 28 | IPI00412498 | Nicotinate phosphoribosyltransferase, Isoform 4 |
| 29 | IPI00549467 | Nit protein 2 |
| 30 | IPI00026546 | Platelet-activating factor acetylhydrolase IB subunit beta |
| 31 | IPI00291922 | Proteasome subunit alpha type-5 |
| 32 | IPI00000783 | Proteasome subunit beta type-8, Isoform 1 |
| 33 | IPI00017526 | Protein S100-P |
| 34 | IPI00219677 | Putative deoxyribose-phosphate aldolase |
| 35 | IPI00016255 | Putative phospholipase B-like1 |
| 36 | IPI00010402 | Putative uncharacterized protein |
| 37 | IPI00009342 | Ras GTPase-activating-like protein IQGAP1 |
| 38 | IPI00010270 | Ras-related C3 botulinum toxin substrate 2 |
| 39 | IPI00291928 | Ras-related protein Rab-14 |
| 40 | IPI00008964 | Ras-related protein Rab-1B |
| 41 | IPI00031169 | Ras-related protein Rab-2A |
| 42 | IPI00014376 | Ras-related protein Rab-31 |
| 43 | IPI00032808 | Ras-related protein Rab-3D |
| 44 | IPI00028481 | Ras-related protein Rab-8A |
| 45 | IPI00020567 | Rho GTPase-activating protein 1 |
| 46 | IPI00026513 | Ribose-5-phosphate isomerase |
| 47 | IPI00010304 | Serpin B10 |
| 48 | IPI00022204 | Serpin B3 |
| 49 | IPI00025318 | SH3 domain-binding glutamic acid-rich-like protein |
| 50 | IPI00788068 | Similar to Arachidonate 5-lipoxygenase |
| 51 | IPI00413293 | Torsin-1A, Isoform 1 |
| 52 | IPI00550917 | Twinfilin-2 |
| 53 | IPI00645078 | Ubiquitin-like modifier-activating enzyme 1 |
| 54 | IPI00453476 | Uncharacterized protein ENSP00000348237 |
| 55 | IPI00016670 | UPF0404 protein C11orf59 |
